# How many child deaths can be averted in Nigeria? Assessing state-level prospects of achieving 2030 sustainable development goals for neonatal and under-five mortality

**DOI:** 10.12688/gatesopenres.12928.1

**Published:** 2019-05-09

**Authors:** Osondu Ogbuoji, Gavin Yamey

**Affiliations:** 1 Center for Policy Impact in Global Health, Duke University, Durham, NC, 27710, USA

**Keywords:** neonatal mortality, under-five mortality, sustainable development goals, Nigeria, sub-Saharan Africa

## Abstract

**Background:** Nigeria’s neonatal mortality rate (NMR) and under-five mortality rate (U5MR) are 39 per 1,000 and 120 per 1,000 live births, respectively. On average, 0.23 million neonates and 0.7 million under-five children die every year, but some states contribute more to this burden than others. If the country is to meet its sustainable development goal (SDG) targets for NMR and U5MR, it needs to make progress at both the national and subnational levels.

**Methods:** Using the 2016-2017 Nigeria Multiple Indicator Cluster Survey (MICS), we estimated state-level neonatal and under-five mortality rates.  Next, we estimated how long it would take for each state to reach the SDG targets for NMR and U5MR. Finally, we estimated the average number of neonatal and under-five deaths that could be averted between 2018 and 2030 in each state under different scenarios.

**Results:** At current average annual rates of decline, Nigeria is unlikely to meet both sustainable development goals targets for NMR and U5MR. At the subnational level, some states are close to or have met both NMR and U5MR targets, while others are projected to meet the targets as late as 2088 (58 years delayed). Between 850,000 and 1.89 million neonatal deaths could be averted between 2018 and 2030, while 3.1 million to 5.96 million under-five deaths could be averted over the same period.

**Conclusions:** Nigeria has the potential to achieve its SDG targets for NMR and U5MR, and in the process avert millions of preventable child deaths. But this will not happen under a business-as-usual approach. The NMR and U5MR trajectories achieved by high-performing states is evidence that achieving these SDG targets is possible. For the country to achieve positive results nationally, systems that encourage peer learning and transfer of technical expertise between states are needed.

## Introduction

In spite of the progress achieved over the past few decades, Nigeria still has very poor child survival metrics ^1, 2^. With an under-five mortality rate (U5MR) of 104 per 1,000 live births, and a neonatal mortality rate (NMR) of 34 per 1,000 live births in 2017, the country ranks 6 ^th^ highest in the world for child mortality, and the highest of all middle-income countries ^3, 4^. It also ranks in the top 10 countries with the highest number of under-five deaths annually ^5^.

Recent estimates suggest that on average about 0.7 million children under the age of five die every year in Nigeria, with about 200,000 of these children dying in the first month of life ^4, 6^. With such a high number of annual neonatal deaths, the country accounted for about 9% of all neonatal deaths in the world in 2016 ^7^. Nigeria and India both account for one-third of all deaths due to lower respiratory infections worldwide; such infections disproportionately affect children under five years ^8^.

In its bid to meet the child mortality target set for the millennium development goals (MDGs), i.e. a two-thirds reduction in the U5MR from 1990 to 2015, Nigeria made significant progress on child mortality. The country reduced the national average U5MR from 213 per 1,000 live births in 1990 to 104 per 1,000 live births in 2015. However, this reduction was not sufficient to meet its MDG under-five mortality target of below 67 per 1,000 live births. Along with other United Nations member states, Nigeria has committed to achieving the 2030 sustainable development goals (SDGs). Target 3.2 of SDG3 calls for all countries to reduce their U5MR to less than 25 deaths per 1,000 live births by 2030, and their NMR to less than 12 deaths per 1,000 live births by 2030.

**Figure 1.**
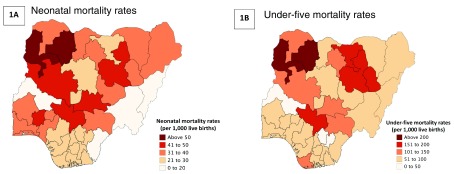
State-level variations in neonatal mortality rates and under-five mortality rates. Source: Authors calculations using MICS 2016–17 data. Maps created with mapchart.net.

Given that Nigeria failed to meet its MDG target on U5MR, early tracking of its progress towards the health-related SDG targets is important—if the country is off track, this would highlight the need for intensified action to accelerate progress. However, while there are already efforts to assess Nigeria’s progress at the country level (e.g., national child mortality is tracked by The UN Inter-agency Group for Child Mortality Estimation), there are three key reasons why it is also critical to assess progress at the subnational level. First, according to latest estimates from the Nigeria Population Commission, the average population of a state in Nigeria is 5.5 million (range: 2.2 million to 13 million), exceeding the population of many countries. Second, there is a large variation in child and neonatal mortality across states in Nigeria (see Figure 1). For NMR, the rate ranges from 21 per 1,000 live births in Adamawa and Akwa-Ibom states to 69 per 1,000 live births in Kano state (the national average is 39 per 1,000 live births). For U5MR, the rate ranges from 45 per 1,000 live births in Kwara state to 210 per 1,000 live births in Zamfara state (the national average is 120 per 1,000) ^9^. An assessment of progress at the state level provides an opportunity to explore these variations in levels and trends. Third, subnational governments (state and local governments) are primarily responsible for healthcare delivery in Nigeria. Therefore, assessing progress at this subnational level has the advantage of providing information that would be useful to policy makers, promote evidence-based decision making, and improve levels of accountability.

In this study, we take advantage of the recently released 2016–2017 Nigeria Multiple Indicator Cluster Survey (MICS) dataset, which for the first time allows state-level estimation of the NMR (SDG 3.2.1) and U5MR (SDG 3.2.2). Nigeria’s MICS is a household survey conducted every five years to assess the health and social protection status of mothers and children around the country. Based on historical trends, we make projections of the feasibility of each state, and of the entire country, attaining the SDG targets for neonatal and under-five mortality by 2030. We also report on the number of child deaths that could potentially be averted under four different scenarios between 2018 and 2030, namely: 1) keeping 2017 mortality rates constant until 2030, 2) allowing mortality rates to change at current trends, 3) allowing mortality rates to decrease at best recorded sub-national rates of decline, and 4) allowing mortality rates to decrease at the rates of decline required to reach SDG targets by 2030.

## Methods

Our analysis involved three major steps. First, we estimated neonatal and under-five mortality rates for each state and for the country. Second, we estimated how long it would take for each state to reach the SDG targets for neonatal and under-five mortality rates. Finally, we estimated the average number of neonatal and under-five deaths that could be averted between 2018 and 2030 in each state under different scenarios.

### i) Estimating neonatal and under-five mortality rates

To estimate state-level neonatal and under-five mortality rates, we analyzed data from the MICS conducted in Nigeria from 2016 to 2017. MICS have been conducted in Nigeria since 1995 by the Nigeria National Bureau of Statistics in concert with the United Nations International Children’s Emergency Fund (UNICEF). These surveys track important indicators of health and social welfare for children, mothers, and their families. Prior to the 2016–17 MICS, previous MICS had estimated child mortality through indirect methods and by using partial birth histories obtained from mothers. The 2016–17 MICS was the first to obtain full birth histories from mothers that would enable more reliable estimates of child mortality using direct estimation methods. Households included in the survey were selected through a multi-stage stratified random sampling. Full birth history information was obtained for every woman present in the sampled household. Details of the survey design and data collection methods have been described by the UNICEF MICS team.

We estimated both neonatal and under-five mortality rates using the full birth histories contained in the 2016–17 MICS datasets. We defined NMR as the probability of a newborn dying in the first 28 days of life, expressed per 1,000 live births, and U5MR as the probability of a child dying before reaching age 5 years, also expressed per 1,000 live births. See Table 1 for details of our NMR and U5MR calculations.

**Table 1.** Equations used for estimates in the study.

	Estimate	Equation	Notes
1	Average annual rates of change (AARC)	$AARC=\frac{Ln\left(\frac{MR_{1}}{MR_{0}}\right)}{t_{1}-t_{0}}$	*MR*_0_ is the mortality rate at the start year, *MR*_1_ is the mortality rate at the end year, AARC is the average annual rate of change (positive for increasing mortality rate, and negative for decreasing mortality rate), *t*_0_ is the start year, and *t*_1_ is the end year.
2	Annual number of births	$Births_{y}=Population_{y}*CBR$	*Births _y_* represent total number of live births in the state for year *i*, and *CBR* represents the crude birth rate. We used the same CBR for all states over the period of estimation
1	Expected mortality rate for each year	$MR_{t}=MR_{t-1{\;}^{\mathrm{aarc}}}$	*MR _t_* is the mortality rate for the index year, *MR*_*t*–1_ is the mortality rate in the previous year, and *aarc* is the average annual rate of change in mortality rate assumed for that scenario.
2	Annual number of under-five deaths for each scenario	$Annual\;Deaths_{s,y}=\sum_{i=0}^{4}x_{y-i}*Cohort\;U5\;Deaths_{y-i}$	*s* represents the scenario, *x* represents the mortality fraction (see Table 8), and *y* represents the index year. *Cohort U*5 *Deaths*_*y*–0_ represents total number of deaths occurring to the birth cohort of the index year, while *Cohort U*5 *Deaths*_*y–i*_ represents the total number of deaths occurring to the birth cohort of *i* years prior to the index year.
3	Total deaths in scenario s for the birth cohort of year y over the first five years of life (i.e. y to y+4)	$Cohort\;U5\;Deaths_{s,y}=U5MR_{s,y}*Total\;Births_{y}$	
4	Under-five mortality rate (U5MR) in scenario s, for year y	$U5MR_{s,y}=U5MR_{s,y-t}*\;e^{aarc_{s}*t}$	*aarc _s_* represents average annual rate of change of mortality rate in scenario *s*, *U*5 *MR* represents under-five mortality rate, and *t* represents continuous time interval

### ii) Measuring progress towards SDG targets for neonatal and under-five mortality rates

For each state, we estimated the required average annual rate of decline (AARD) needed for the state to achieve the SDG neonatal mortality target of 12 deaths per 1000 live births and the SDG under-five mortality target of 25 deaths per 1000 live births.

To assess if states were on track to meet their SDG targets for NMR and U5MR, we calculated historical trends in AARD for each state using mortality rate estimates reported in the Nigeria Demographic and Health Surveys (NDHS) of 2003, 2008, and 2013. Like the 2016–17 MICS (and unlike previous MICS), the previous NDHS used direct estimation of child mortality through full birth histories so it was possible to compute trends over the timeframe of interest. We calculated separate estimates for NMR and U5MR for five-year periods (2003 to 2008, and 2008 to 2013), with the assumption that mortality rates changed using exponential decay over the period. Then, we applied the annual rate of decline for each state to the its 2017 mortality rate estimates to project how long it would take for that state to achieve its SDG targets for NMR and U5MR. Since state-level estimates were not available prior to 2011, we calculated AARDs for each of the six regions in the country using equation 1 in Table 1, and applied the regional AARD to all the states in the region.

### iii) Quantifying avertable deaths between 2018 to 2030

To quantify the number of avertable deaths between 2018 and 2030 from potential improvements in neonatal and under-five mortality, we applied our projections of state-level mortality rate estimates to state-level population projections from the Nigeria National Population Commission for four different scenarios and computed the difference.

First, we modelled a baseline “do nothing” (or “business as usual”) scenario, where we assumed that NMR and U5MR do not change from 2017 levels, i.e., they remain unchanged between 2017 and 2030. The second, “historical trends” scenario assumed that states will experience AARD trends similar to the trends they experienced in the period leading up to the MDGs. In this scenario, each state starts out with its NMR and U5MR for 2017, and then experiences an annual reduction in both mortality rate estimates between 2018 and 2030 based on its historical AARD estimates. In our third, “best in country” scenario, we assumed that between 2018 and 2030, each state in the country would attain the AARD observed in the best performing zone during the years leading up to the MDGs. Finally, we modeled an “SDG target” scenario where we assumed that between 2018 and 2030, each state will experience the AARD that is required for it to achieve the NMR and U5MR SDG targets by 2030.

In all four scenarios, we estimated the number of neonatal and under-five deaths that would occur for each year between 2018 and 2030 using mortality fractions (see Table 2) and estimates of average annual rates of change in mortality rates (see Table 3). Deaths averted were then calculated by subtracting the number of deaths from each scenario from the number of deaths calculated for the “do nothing” scenario.

**Table 2.** Proportion of under-five deaths occurring in each of the first five years of life.

Period	Number of deaths	Percent of total
Year 1	3,047	52%
Year 2	1,330	23%
Year 3	1,004	17%
Year 4	349	6%
Year 5	145	2%
**Total**	**5,875**	**100%**

Source: Authors calculations using MICS 2016–2017 data of all under-five deaths reported in the five years prior to the survey.

**Table 3.** Average Annual Rates of Change (AARC) between 2008 and 2013 computed from Nigeria Demographic and Health Surveys.

	NMR			U5MR		
Geo-political zone	AARC (2003 – 2013)	AARC (2008 – 2013)	Best rate	AARC (2003 – 2013)	AARC (2008 – 2013)	Best rate
National	-2.6	-1.4	-2.6	-4.5	-4.0	-4.5
North-Central	-4.2	-2.9	-4.2	-5.0	-5.9	-5.9
North-East	-3.3	-4.0	-4.0	-4.8	-6.5	-6.5
North-West	-2.4	-1.4	-2.4	-3.7	-3.2	-3.7
South-East	0.9	-6.3	-6.3	2.5	-3.1	-3.1
South-South	-5.0	-8.0	-8.0	-6.6	-8.4	-8.4
South-West	0.0	1.3	0.0	-2.3	0.2	-2.3

Source: Authors calculations using datasets from Nigeria Demographic and Health Surveys 2003, 2008, and 2013. Notes: 1. Average Annual Rate of Change (AARC) was calculated using the formula: AARC=(MRendMRstart)End−Start∗100. Positive values indicate rising neonatal mortality rates while negative values indicate falling neonatal mortality rates.

Finally, we concluded our analysis by ranking states based on their NMR (SDG 3.2.1) and U5MR (SDG 3.2.2) estimates in 2017. We also rank states based on the estimated annual number of neonatal and under-five deaths they have, in order to identify the states that account for most of the burden of neonatal and under-five mortality in Nigeria.

Mortality rates estimates were computed using Stata software version 15.1 and we used Microsoft Excel for Mac version 16.22 to make projections. An expanded description of methods applied is available as Extended data ^10^.

## Results

### i) Neonatal mortality rates and progress towards SDG 3.2.1

Table 4 shows our estimates of the national and state NMRs from MICS 2016–2017. The estimated national NMR in 2017 was 37 per 1,000 live births (95% CI: 36-39). But at the state level, NMR ranged from 12 per 1,000 live births (95% CI: 7-22) in Kwara state to 65 per 1,000 live births (95% CI: 59-72) in Kano state.

**Table 4.** State-level trends in neonatal mortality rates (NMR) and progress towards achieving sustainable development goals (SDG) target for NMR.

			SDG scenarios			
			At current AARC (2003–2013)		At best in-country AARC (2003–2013)	
State	Neonatal mortality rate (2017)	Required AARC to reach SDG target	Year SDG target will be met	Number of years delay beyond 2030	Year SDG target will be met	Number of years delayed
**North-Central**						
Benue	34 (25, 45)	-8	2042	12	2030	0
FCT Abuja	23 (17, 32)	-5	2033	3	2025	3 years early
Kogi	39 (29, 53)	-9	2045	15	2032	On track
Kwara	12 (7, 22)	0	2018	SDG target met	2017	SDG target met
Nasarawa	47 (39, 58)	-11	2050	20	2034	4
Niger	50 (42, 60)	-11	2051	21	2035	5
Plateau	39 (31, 49)	-9	2045	15	2032	On track
**North-East**						
Adamawa	20 (14, 29)	-4	2030	1 year early	2024	7 years early
Bauchi	41 (33, 49)	-9	2047	17	2032	2
Borno	30 (20, 45)	-7	2040	10	2029	1 year early
Gombe	32 (25, 41)	-7	2041	11	2029	1 year early
Taraba	17 (11, 26)	-3	2026	4 years early	2022	2 years early
Yobe	39 (31, 49)	-9	2046	16	2032	2
**North-West**						
Jigawa	30 (24, 38)	-7	2056	26	2029	On track
Kaduna	29 (22, 38)	-7	2054	24	2028	2 years early
Kano	65 (59, 72)	-13	2088	58	2038	8
Katsina	37 (30, 45)	-9	2064	34	2031	1
Kebbi	44 (37, 54)	-10	2072	42	2033	3
Sokoto	40 (32, 49)	-9	2068	38	2032	On track
Zamfara	52 (45, 62)	-11	2079	49	2035	5
**South-East**						
Abia	30 (21, 44)	-7	2032	2	2029	On track
Anambra	21 (13, 32)	-4	2026	3 years early	2024	5 years early
Ebonyi	22 (14, 33)	-5	2026	4 years early	2024	1 year early
Enugu	23 (15, 37)	-5	2028	2 years early	2025	5 years early
Imo	29 (20, 42)	-7	2031	1	2028	1 year early
**South-South**						
Akwa Ibom	26 (19, 37)	-6	2027	3 years early	2027	3 years early
Bayelsa	34 (25, 46)	-8	2030	On track	2030	On track
Cross River	18 (11, 29)	-3	2022	8 years early	2022	8 years early
Delta	28 (20, 41)	-7	2028	2 years early	2028	2 years early
Edo	24 (21, 29)	-5	2026	4 years early	2026	4 years early
Rivers	25 (16, 39)	-6	2026	4 years early	2026	4 years early
**South-West**						
Ekiti	38 (26, 57)	-9			2032	2
Lagos	35 (28, 45)	-8	On average, neonatal mortality rates were static or rising every year in the south-west zone between 2003 and 2013.		2031	2 years early
Ogun	32 (22, 47)	-8			2029	2 years early
Ondo	32 (22, 46)	-8			2029	On track
Osun	49 (35, 69)	-11			2035	5
Oyo	38 (28, 53)	-9			2032	2
**National**	37 (36, 39)	**-9**	**2060**	**30**	**2031**	**1**

Notes: 1. Neonatal mortality rate (NMR) was defined as the probability that a who was born alive dies in the first month of life, expressed per 1,000 live births. 2. Average Annual Rate of Change (AARC) was calculated using the formula: AARC=(NMRendNMRstart)End−Start∗100. Positive values indicate rising neonatal mortality rates while negative values indicate falling neonatal mortality rates. 3. Sustainable Development Goals (SDG) target for neonatal mortality rate is 12 deaths per 1000 live births by 2030.

For Nigeria to achieve its SDG target for NMR of 12 deaths per 1,00 live births (SDG 3.2.1), its national NMR will have to decrease by 9% every year, on average, between 2018 and 2030. However, the AARD required to reach SDG 3.2.1 at the state level varies widely. While Kwara state has already achieved the SDG 3.2.1 target, some other states such as Zamfara and Kano will need to achieve an average annual decrease in NMR of 11%, and 13% respectively, in order to meet the NMR SDG targets.

Our estimates of historical AARD in NMR ranged between 1.4% per year and 2.6% per year at the national level. At the subnational level, the AARD for NMR varied across zones. The South-South zone of the country recorded the largest AARD of 8% per year between 2008 and 2013, while the South-West zone of the country showed worsening NMR in the same period with an increase in NMR 1.3% per year (see Table 3).

**Figure 2.**
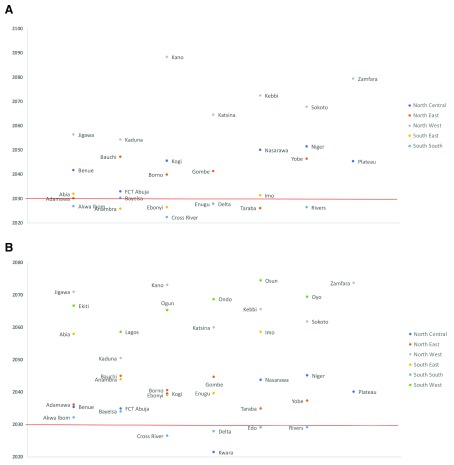
**A**. Year by which each Nigerian state will achieve the sustainable development goals target for neonatal mortality rate (SDG 3.2.1) of 12 per 1,000 live births assuming states maintain recent average annual rates of change. **B**. Year by which each state will achieve the sustainable development goals target for under-five mortality rate (SDG 3.2.2) of 25 per 1,000 live births assuming states maintain recent average annual rates of change. States below the red line are on track to reach their SDG targets by 2030 while states above the red line ar not on track to reach their SDG targets.

At historical AARDs, Nigeria, as a country, will achieve its SDG target for NMR in 2060, 30 years behind the SDG target year of 2030. However, progress across the different states would vary. At historical rates of decline, 12 out of 37 states would have met or surpassed the SDG NMR target by 2030, while 19 states will achieve the SDG NMR target between 2031 (1 year later) and 2088 (58 years later). We did not estimate target years for the five states in the South-West zone of the country because, unlike other zones, the South-West zone showed *increases* in NMR between 2003 and 2013 that ranged from 0.3% per year to 1.3% per year. As such, they would be moving away, not towards, the SDG NMR target (See Table 4, and Figure 2A).

However, if between 2018 and 2030, each state maintains an AARD in NMR equal to the best historical rate of decline in the country in the MDG era (i.e. 8% decline per year), Nigeria will achieve its SDG target for NMR in 2031 (1 year behind schedule). In this scenario, 26 out of 37 states will achieve the NMR target for the SDGs while 11 will not. The years in which states will reach NMR targets for SDG range from 2022 (8 years early) to 2038 (8 years behind schedule).

### ii) Under-five mortality rates and progress towards SDG 3.2.2

At the national level, U5MR in 2017 was 118 per 1,000 live births (95% CI: 115-121), but at the state level, U5MR ranged from 32 per 1,000 live births (95% CI: 23-46) in Kwara state, to 209 per 1,000 live births (95% CI: 193-226) in Zamfara state. Consequently, Nigeria will need to maintain a national AARD in U5MR of 12% per year between 2018 and 2030 in order to meet the SDG target for U5MR of 25 deaths per 1,000 live births (SDG 3.2.2). To achieve SDG 3.2.2 targets at state level, states will have to maintain an AARD that ranges from 2% per year in Kwara state to 16% per year in Kano, Jigawa, and Zamfara states ( Table 5).

**Table 5.** State-level trends in under-five mortality rates (U5MR) and progress towards achieving sustainable development goals (SDG) target for U5MR.

			Sustainable Development Goals (SDG) scenarios			
			At current AARC (2003–2013)		At best in-country AARC (2003–2013)	
State	Under-five mortality rate (2017)	Required AARC to reach SDG target	Year SDG target will be met	Number of years delay beyond 2030	Year SDG target will be met	Number of years delayed
**North-Central**						
Benue	73 (60, 90)	-8	2035	5	2030	On target
FCT Abuja	71 (59, 87)	-8	2035	5	2030	On target
Kogi	93 (76, 114)	-10	2039	9	2033	3
Kwara	32 (23, 46)	-2	2021	-9	2020	10 years early
Nasarawa	120 (105, 137)	-12	2044	14	2036	6
Niger	129 (115, 145)	-13	2045	15	2037	7
Plateau	97 (83, 112)	-10	2040	10	2033	3
**North-East**						
Adamawa	87 (73, 103)	-10	2036	6	2032	2
Bauchi	154 (139, 170)	-14	2045	15	2039	9
Borno	115 (94, 142)	-12	2040	10	2035	5
Gombe	151 (134, 169)	-14	2045	15	2038	8
Taraba	80 (65, 98)	-9	2035	5	2031	1
Yobe	93 (80, 109)	-10	2037	7	2033	3
**North-West**						
Jigawa	189 (172, 206)	-16	2071	41	2041	11
Kaduna	87 (75, 102)	-10	2050	20	2032	2
Kano	205 (193, 217)	-16	2073	43	2042	12
Katsina	125 (111, 139)	-12	2060	30	2036	6
Kebbi	155 (140, 171)	-14	2066	36	2039	9
Sokoto	134 (119, 150)	-13	2062	32	2037	7
Zamfara	209 (193, 226)	-16	2074	44	2042	12
**South-East**						
Abia	88 (70, 111)	-10	2058	28	2032	2
Anambra	57 (43, 76)	-6	2044	14	2027	3 years early
Ebonyi	49 (36, 66)	-5	2039	9	2025	5 years early
Enugu	50 (33, 64)	-5	2040	10	2025	5 years early
Imo	89 (72, 111)	-10	2058	28	2032	2
**South-South**						
Akwa Ibom	88 (72, 107)	-10	2032	2	2032	2
Bayelsa	103 (87, 124)	-11	2034	4	2034	4
Cross River	55 (42, 72)	-6	2026	4 years early	2026	4 years early
Delta	62 (48, 80)	-7	2028	2 years early	2028	2 years early
Edo	68 (62, 76)	-8	2029	1 year early	2029	1 year early
Rivers	68 (52, 90)	-8	2029	1 year early	2029	1 year early
**South-West**						
Ekiti	76 (57, 101)	-9	2067	37	2030	On target
Lagos	64 (53, 77)	-7	2059	29	2028	2 years early
Ogun	74 (57, 96)	-8	2065	35	2030	2 years early
Ondo	80 (63, 102)	-9	2069	39	2031	1
Osun	91 (70, 117)	-10	2074	44	2032	2
Oyo	81 (64, 102)	-9	2069	39	2031	1
**National**	118 (115, 121)	**-12**	**2052**	**22**	**2036**	**6**

Notes: 1. Under-five mortality rate (U5MR) was defined as the probability that a child who was born alive dies in the first five years of life, expressed per 1,000 live births. 2. Average Annual Rate of Change (AARC) was calculated using the formula: AARC=(U5MRendU5MRstart)End−Start∗100. Positive values indicate rising under-five mortality rates while negative values indicate falling under-five mortality rates. 3. Sustainable Development Goals (SDG) target for under-five mortality rate is 25 deaths per 1000 live births by 2030.

Historical estimates of AARDs for U5MR ranged from 4.0% to 4.5% per year at the national level. At the subnational level, AARD for U5MR was highest in the South-South zone (at 8.4% per year), but showed reversals in the South-East zone with rates of increase as high as 2.5% per year (see Table 3).

Our model suggests that Nigeria is not on track to meet the SDG target for U5MR (SDG 3.2.2) by 2030. At current national AARDs, it will achieve SDG 3.2.2 in 2052 (22 years behind schedule). At the subnational level, 5 out of 37 states will meet or surpass the SDG 3.2.2 target on or before 2030, while the other 32 states are not on track to achieve SDG 3.2.2 target by 2030. The years in which states will reach U5MR targets for SDG range from 2021 (9 years ahead of schedule) in Kwara state to 2074 (44 years behind schedule) in Zamfara and Osun states (see Table 5 and Figure 2B).

If all states maintained an AARD in U5MR equal to the best performing in country (i.e. 8.4% decline per year), Nigeria will still not meet its U5MR target by 2030, but would do so by 2036 (six years behind schedule). In this scenario, 13 out of 37 states will meet or surpass their targets for U5MR by 2030, while the other 24 will not. The years in which states will reach U5MR targets for SDG range from 2020 (10 years early) in Kwara state to 2042 (12 years behind schedule) in Kano and Zamfara states (see Table 5).

### iii) Avertable neonatal and under-five deaths between 2018 and 2030

**Figure 3.**
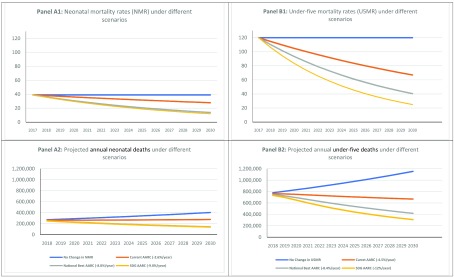
Annual estimates of neonatal mortality rates, neonatal mortality counts, under-five mortality rates, and under-five mortality counts between 2018 and 2030.

We estimated the number of neonatal and under-five deaths that would occur between 2018 and 2030 under four different scenarios described in the Methods section above (sub-section on quantifying avertable deaths between 2018 and 2030). Figure 3 shows trends in mortality rates and number of annual deaths for each of the four scenarios over the period 2018 to 2030. In the “do nothing” scenario, in which each state maintains its 2017 NMR and U5MR throughout the period 2018 to 2030, a total of 4.3 million neonatal deaths and 12.4 million under-five deaths would occur between 2018 and 2030 (see Table 6). This number of deaths translates to an average of 330,000 neonatal deaths per year and 950,000 under-five deaths per year between 2018 and 2030. In the second scenario, the “historical trends” scenario, in which we applied the historical rates of decline for each zone to all states within the zone, there would be an estimated 3.5 million neonatal deaths and 9.3 million under-five deaths between 2018 and 2030. In the third scenario, the “best in country” scenario, in which every state would attain the AARD observed by the best performing in country, there would be an estimated 2.5 million neonatal deaths and 7.5 million under-five deaths between 2018 and 2030. In our last scenario, the “SDG target” scenario, in which each state will maintain from 2018–2030 the AARD required to reach the SDG targets for NMR and U5MR by 2030, there would be an estimated 2.4 million neonatal deaths and 6.4 million under-five deaths over this time period.

**Table 6.** Projected number of neonatal and under-five deaths between 2018 and 2030 if current mortality rates persist.

	Total deaths (2018 to 2030)	Average annual deaths (2018 to 2030)	2018 to 2020	2021 to 2025	2025 to 2030
Neonatal deaths	4,332,601	333,277	842,948	1,601,587	1,888,066
Under-five deaths	12,408,845	954,527	2,415,162	4,587,492	5,406,190

Comparing each scenario with the baseline “do nothing” scenario, we estimate that if states maintain historical AARDs, at least 850,000 neonatal deaths will be averted between 2018 and 2030 while 3.1 million or more under-five deaths will be averted in the same period compared to the baseline. However, if every state achieves and maintains the best AARD achieved during the MDG era, at least 1.83 million neonatal deaths and 4.9 million under-five deaths would be averted between 2018 and 2030. In the event that each state in Nigeria achieves and maintains the AARD required for it to meet its SDG targets for NMR and U5MR, we estimate that compared to the baseline scenario, at least 1.89 million neonatal deaths and 5.96 million under-five deaths will be averted between 2018 and 2030 ( Table 7).

**Table 7.** Number of neonatal and under-five deaths averted between 2018 and 2030 under different scenarios.

	Projected total number of deaths (2018 to 2030)				Projected number of deaths averted over baseline (2018 to 2030)		
	(1)	(2)	(3)	(4)	(5)	(6)	(7)
Age	No change in mortality rate (Baseline)	Assuming current AARC trend	Best in-country AARC	SDG track	Current AARC trends (2) - (1)	Best in-country AARC (3) - (1)	AARC required for SDG (4) - (1)
Neonatal mortality	4,332,601	3,480,237	2,497,842	2,440,131	852,364	1,834,759	1,892,470
Under-five mortality	12,408,845	9,301,971	7,454,166	6,449,871	3,106,874	4,954,678	5,958,973

Notes: 1. Average Annual Rate of Change (AARC) was calculated using the formula: AARC=(MRendMRstart)End−Start∗100. Positive values indicate rising neonatal mortality rates while negative values indicate falling neonatal mortality rates. 2. The “No Change” scenario assumes that the mortality rate observed in 2018 for each sub-national entity would be maintained at the same level between 2018 and 2030 (i.e. AARC = 0). 3. The “Current AARC” scenario assumes that mortality rates for each sub-national entity continue to change between 2018 and 2030, at the AARC observed in the most recent five years for which data is available. 4. The “Best In-Country” scenario assumes that all subnational entities achieves AARCs between 2018 and 2030, that are similar to that achieved by the best performing sub-national entity in the most recent five years for which data is available. 5. The “AARC for SDG” scenario assumes that between 2018 and 2030, each sub-national entity will achieve the AARC it requires in order to achieve the Sustainable Development Goals (SDG) target for that mortality rate.

Our models suggest that even if Nigeria achieves its SDG targets for NMR of 12 per 1,000 live births by 2030, there would still be at least 139,000 neonatal deaths per year if the NMR remains at the SDG target rate with no further improvement. In the same vein, if Nigeria achieves its SDG targets for U5MR of 25 per 1,000 live births, we project that on average, there would still be at least 308,000 under-five deaths per year if the U5MR remains at the SDG target rate with no further improvement.

### iv) State performance and concentration of the burden of mortality

We ranked states based on their most recent levels of NMR and U5MR, as well as their contributions to the annual number of neonatal and under-5 deaths. Table 8 lists the states in the top and bottom performance quintiles measured by mortality rates, while Table 9 lists the states that account for the largest portion of neonatal and under-five mortality based on 2018 estimates.

**Table 8.** Best and worst performing states for neonatal and under-five mortality rates.

Best performing states (Top 20%, by 2017 mortality rate estimates)					
State	Neonatal mortality rate	Rank	State	Under-five mortality rate	Rank
Kwara	12	1	Kwara	32	1
Taraba	17	2	Enugu	46	2
Cross River	18	3	Ebonyi	49	3
Adamawa	20	4	Cross River	55	4
Anambra	21	5	Anambra	57	5
Ebonyi	22	6	Delta	62	6
FCT Abuja	23	7	Lagos	64	7
Worst performing states (Bottom 20%, by 2017 mortality rate estimates)					
State	Neonatal mortality rate	Rank	State	Under-five mortality rate	Rank
Bauchi	41	31	Sokoto	134	31
Kebbi	44	32	Gombe	151	32
Nasarawa	47	33	Bauchi	154	33
Osun	49	34	Kebbi	155	34
Niger	50	35	Jigawa	189	35
Zamfara	52	36	Kano	205	36
Kano	65	37	Zamfara	209	37

**Table 9.** States with the highest neonatal and under-five mortality burden.

States with the highest burdens of neonatal and under-five mortality					
State	Estimated number of neonatal deaths in 2018	% of national neonatal deaths	State	Estimated number of under-five deaths in 2018	% of national under-five deaths
Kano	34,200	13%	Kano	107,800	14%
Lagos	17,800	7%	Jigawa	43,900	6%
Oyo	12,100	4%	Bauchi	40,500	5%
Katsina	11,600	4%	Katsina	39,000	5%
Niger	11,300	4%	Zamfara	37,900	5%
Bauchi	10,700	4%	Lagos	31,300	4%
Kaduna	9,600	4%	Niger	28,200	4%

States performing in the top quintile for NMR include Kwara, Taraba, Cross River, Adamawa, Anambra, Ebonyi, and the Federal Capital Territory, with NMRs between 12 and 23 per 1,000 live births in 2017. States performing in the bottom quintile for NMR include Bauchi, Kebbi, Nasarawa, Osun, Niger, Zamfara, and Kano, with NMRs between 41 and 65 per 1,000 live births in 2017.

For U5MR, the states performing in the top quintile include Kwara, Enugu, Ebonyi, Cross River, Anambra, Delta, and Lagos, with U5MRs between 32 and 64 per 1,000 live births in 2017. By contrast, states performing in the bottom quintile for U5MR include Sokoto, Gombe, Bauchi, Kebbi, Jigawa, Kano, and Zamfara, with U5MR between 134 and 209 per 1,000 live births in 2017.

Our model shows that Kano state accounts for 13% of projected national neonatal mortality in 2018 with an estimated 34,000 neonatal deaths, and 14% of projected national under-five mortality in 2018 with an estimated 108,000 under-five deaths. In sum, only a small number of states account for about 40% of total neonatal and under-five deaths in 2018. They include Kano, Lagos, Oyo, Katsina, Niger, Bauchi, and Kaduna states for neonatal mortality, and Kano, Jigawa, Bauchi, Katsina, Zamfara, Lagos, and Niger for under-five mortality (see Table 9 for details)

## Discussion

### Statement of principal findings

Based on recent survey data and analysis of historical trends, we modeled future trends in state-level neonatal and under-five mortality in Nigeria between 2018 and 2030. Our findings show that if historical trends continue, Nigeria as a country, and multiple states within the country, are unlikely to meet the SDG targets for neonatal and under-five mortality by 2030. While some states have already attained or will attain the SDG targets several years before 2030, others are projected to attain the SDG targets as late as 2088 (58 years behind schedule).

We also show that if current mortality rates remain unchanged, up to 4.3 million neonatal deaths and 12.5 million under-five deaths are likely to occur in Nigeria between 2018 and 2030. However, if each state attains the required annual rate of decline needed to achieve the SDG targets for neonatal and under-five mortality, 1.9 million neonatal deaths and 5 million under-five deaths can be averted between 2018 and 2030.

Another important finding from this study is that if Nigeria only meets the SDG targets and does not surpass them—in other words, if it only achieves a U5MR of 25 deaths per 1,000 live births and an NMR of 12 deaths per 1,000 live births by 2030—the country would still have a high burden of avertable mortality. In a scenario in which it reaches these two SDG targets, at least 139,000 neonatal deaths and 308,000 under-five deaths would still occur every year in Nigeria. This annual number of deaths translates to more than 800 under-five deaths per day.

### Comparison with other estimates

Our estimates are consistent with the 2016 estimates of the NMR and U5MR in Nigeria from the Global Burden of Disease (GBD) study. The 2016 GBD estimated an NMR for Nigeria of 35.7 per 1,000 live births (95% CI: 28.8-43.5) for NMR and a U5MR of 108.7 per 1,000 live births (95% CI: 93.0- 126.2) ^6^.

In addition, our projections are consistent with projections from three other groups: (a) the UN Inter-agency Group for Child Mortality Estimation (UN-IGCME) ^11^, (b) the GBD 2016 SDG collaborators ^12^, and (c) McArthur and colleagues ^13^. All three studies found that if current trends persist, Nigeria will not reach its SDG targets for child mortality by 2030.

However, our study goes a step further to provide state-level estimates and assessment of states’ performance. Our estimates are also consistent with those of Liu and colleagues, who showed that by 2030, Nigeria would account for up to 7% the estimated 4.4 million annual child deaths in 2030 ^14^.

### Strengths and weaknesses of the study

Our study contributes to the current literature in important ways. To the best of our knowledge, this is the first study to make subnational projections of neonatal and under-five mortality in Nigeria. Sub-national modeling is important because primary health care is mostly managed at the state and local government levels in Nigeria, not at the federal government. Our finding of a large disparity in mortality rates between states shows that they have performed very differently in adopting and scaling up child survival policies, thus we hope that our state level analysis will be helpful to policymakers. The lack of state-level estimates and projections make it difficult for policy makers at the state level to track their performance or make informed decisions about the future effects of certain health policies.

Our study uses the recently released MICS 2016–2017 household survey data, which has a number of advantages. This is the most recent household survey conducted in Nigeria, it has both national and state-level estimates for neonatal and under-five mortality, and it is the only one conducted in the post-2015 SDG era. Therefore, our analysis provides a good baseline for policy making.

Another strength of our study is that we adopted reasonable assumptions of rates of change. While we could have chosen to model scenarios with the rates of change of the best performing countries outside Nigeria, we decided to keep our analysis grounded in historical evidence. We therefore restricted our analysis of scenarios to rates of decline that have been achieved within Nigeria, and compared them to what will need to be achieved for the SDG targets to be met.

One important limitation of our study is that although we show differences in state-level performance for both neonatal and under-five mortality, we do not analyze the reasons for these differences. So, our study has not shown *why* some states perform better than others or *which policies* might have improved their performance. This is an important subject for future research.

### Implications for policy makers

The wide variation in performance in neonatal and under-five mortality seen across states in this study creates an opportunity for states to learn from each other. While a state like Kwara has already met the SDG target for NMR, other states like Zamfara and Kano will only meet their SDG targets for NMR around 2079 (a 49-year delay) and 2088 (a 58-year delay) respectively, if their current rates of decline are maintained. For under-five mortality, while Kwara state is projected to reach its U5MR targets for SDG3 around 2021 (9 years ahead of schedule), others like Zamfara and Osun states are projected to reach their U5MR targets for SDG3 by 2074 (44 years behind schedule).

Highlighting these differences is only a first step. Opportunities are needed for greater dialogue among policymakers within the different states. These dialogues could take the form of study visits to states performing in the top quintile for neonatal mortality (Kwara, Taraba, Cross River, Adamawa, Anambra, Ebonyi, and the Federal Capital Territory) and under-five mortality (Kwara, Enugu, Ebonyi, Cross River, Anambra, Delta, and Lagos states). They could also take the form of collaborative learning networks, or joint programming. Fostering these dialogues will encourage partnerships, stimulate homegrown innovation, and create an environment where evidence-based learning and decision making becomes the norm. One model for this kind of shared learning is the Joint Learning Network for Universal Health Coverage.

Another important policy implication of this study is that it highlights the importance of not just tracking mortality rates, but also tracking rates of change. While current mortality rates create a snapshot of performance at the time of the survey, it is impossible for policy makers to project how much progress is possible or feasible without paying attention to rates of change. For example, while neonatal mortality rates in the south-west region of the country were better than other regions between the period 2003 and 2013, they ended the period with NMR worse than at the beginning because of the rates of change they experienced. By contrast, the north-west and north-east zones have the highest neonatal mortality rates by recent estimates, but over time they have shown some progress in the form of a downward trend.

The importance of rates of decline are further seen in the modeling of different scenarios in our study. For example, if every state within the country achieves an AARD equal to the best performers (8% per year for NMR, and 8.4% per year for U5MR), a total of 1.8 million neonatal or 4.9 million under-five deaths would be averted between 2018 and 2030. If, on the other hand, each state achieves the AARD required to meet its SDG3 target (between 0% and 13% for NMR, and between 2% and 16% per year for U5MR), a total of 1.9 million neonatal deaths or 5.9 million under-five deaths could be averted between 2018 and 2030. However, achieving such high rates of decline will require sustained efforts on the part of policy makers. For comparison, in the period between 2006 and 2016, Rwanda, with all its investments in health reform, recorded an average rate of decline in under-five mortality of 9.2% per year ^13^. Our study found that the best rates of decline achieved in Nigeria were 8% per year for NMR and 8.4% per year for U5MR over a 10-year period from 2003 to 2013. Furthermore, long-run estimates of average rates of decline in U5MR for Nigeria between 1990 and 2016 (a 26-year period) were between 2.7% and 3.3% per year ^6, 7^. Therefore, a policy maker seeking to achieve an AARD of up to 16% in Nigeria should be prepared to make the required investments and sustain these investments over the long-run. This would require innovation, resources, persistence, and political will.

Finally, although our study did not analyze the reasons for variation across states, there is sufficient evidence in the literature on interventions that can reduce neonatal and under-five mortality in low- and middle-income countries. From recent estimates, the main causes of under-five mortality in Nigeria include malaria, diarrheal diseases, neonatal encephalopathy, lower respiratory tract infections, and neonatal preterm births ^5, 15^. These are all conditions for which there are simple, low-cost, lifesaving interventions ^16– 18^. Reliable platforms also exist to deliver these interventions ^18– 20^ and are captured in the Global Strategy for Women’s, Children’s and Adolescent Health (2016–2030) ^21^.

Even though there are wide variations in mortality rates exist across the country, there is evidence that the main causes of child mortality do not vary significantly across the country. Uthman and colleagues conducted an exploratory spatial analysis of under-five mortality in Nigeria using data from the 2008 Nigeria Demographic and Health Survey, and concluded that the “vast majority of states (73%)” do not show evidence of special-cause variation in under-five mortality ^22^. Therefore, there is potential for remarkable progress by addressing the common causes.

## Conclusion

Although estimates from our study and several others suggest that Nigeria will not meet its SDG targets for NMR and U5MR at current rates of decline, we show that there is wide variation at the state level with some states already at SDG3 targets and others projected to experience delays of up to 58 years. These variations create opportunities for low-performing states to learn from high-performing states. As Nigeria moves towards achieving its child survival targets, it will be critical for policymakers at the state-level to track their performance on both SDG targets for NMR (SDG 3.2.1) and U5MR (SDG 3.2.2), measure their performance against the policies they introduce, and continuously assess what is possible by comparing their performance with high performing states. Doing this will help avert millions of child deaths in Nigeria between 2018 and 2030.

## Data availability

### Source data

All datasets used in this study (Nigeria 2016-07 Survey) are publicly available at https://dhsprogram.com/ and http://mics.unicef.org/surveys. Interested readers can access the data by filling out an online form describing the intended use of the data. Once approved, data can be downloaded from the respective sites. The online form is accessible following clicking to download the file via the MICS site.

### Extended data

Open Science Framework: Supplementary data: How many child deaths can be averted in Nigeria? Assessing state-level prospects of achieving 2030 sustainable development goals for neonatal and under-five mortality. https://doi.org/10.17605/OSF.IO/8QMKE ^10^

This project contains the following extended data:

- Supplement for -- State_level_trends_in_maternal_and_child_mortality_in_Nigeria_25MAR2019_Final.pdf (Expanded methods and results)

Data are available under the terms of the Creative Commons Zero “No rights reserved” data waiver (CC0 1.0 Public domain dedication).
