# Supplementary material for: How many child deaths can be averted in Nigeria? Assessing state-level prospects of achieving 2030 sustainable development goals for neonatal and under-five mortality
Source: Gates Open Res. 2019 May 9;3:1460. [Version 1] doi: 10.12688/gatesopenres.12928.1 (PMC6773975; doi:10.12688/gatesopenres.12928.1)
Supplement: Peer review history

# Peer review: Reviewer response for version 1

**DOI:** 10.21956/gatesopenres.14030.r27674

**Published:** 2019-09-30

**Authors:** Seungman Cha

**Affiliations:** 1 London School of Hygiene & Tropical Medicine, London, UK

**Baseline scenario**

 I think that the baseline scenario should be the mortality rate based on historical trends rather than an unchanging MR. I would suggest the authors to re-describe discussion parts particularly in relation to the key findings on the basis of comparison between the scenario 2 and scenario 4.

**Novelty of the estimation methods**

 Measuring progress towards SDG targets for neonatal and under-five mortality rates based on different set of scenarios such as historical trends, worst and best case, and SDG target scenario has been applied in several studies. Although some of these studies were done at the global level, these studies should be referenced since the methodology particularly with regards to the four types of scenario is not novel. (For instance. You *et al., *2015 ^1^; Alkema *et al., *2016 ^2^)

 In addition, the study done by Nick Golding ^3^ should be stated in the background, particularly regarding its limitation and needs for another study in Nigeria. 

 The fact that there exists enormous inequality in U5MR, NMR and its progress by province/state has been emphasized in a number of papers and UN reports. I hope that the authors could add at least one paragraph about the possible explanations for the remarkable progress in certain provinces of Nigeria, which will make the paper more attractive to the potential readers working in other countries with similar context.

**References**

 Some of the references should be updated or added. For instance, Global Burden of Disease 2018 publications.

 Can the authors add a legend for X-axis for Figure 2?

 Overall, this paper contains important findings and is thus worth to be published.

I confirm that I have read this submission and believe that I have an appropriate level of expertise to confirm that it is of an acceptable scientific standard, however I have significant reservations, as outlined above.

## References

[1] : Global, regional, and national levels and trends in under-5 mortality between 1990 and 2015, with scenario-based projections to 2030: a systematic analysis by the UN Inter-agency Group for Child Mortality Estimation. *The Lancet*.2015;386(10010) : 10.1016/S0140-6736(15)00120-8 2275-2286 10.1016/S0140-6736(15)00120-8 26361942

[2] : Global, regional, and national levels and trends in maternal mortality between 1990 and 2015, with scenario-based projections to 2030: a systematic analysis by the UN Maternal Mortality Estimation Inter-Agency Group. *Lancet*.2016;387(10017) : 10.1016/S0140-6736(15)00838-7 462-74 10.1016/S0140-6736(15)00838-7 26584737PMC5515236

[3] : Mapping under-5 and neonatal mortality in Africa, 2000-15: a baseline analysis for the Sustainable Development Goals. *Lancet*.2017;390(10108) : 10.1016/S0140-6736(17)31758-0 2171-2182 10.1016/S0140-6736(17)31758-0 28958464PMC5687451

# Peer review: Reviewer response for version 1

**DOI:** 10.21956/gatesopenres.14030.r27197

**Published:** 2019-06-18

**Authors:** Roy Burstein

**Affiliations:** 1 Institute for Disease Modeling (IDM), Bellevue, WA, USA

The authors address the important issue of subnational variation in under-5 mortality. They produce subnational estimates of neonatal and under-5 mortality for all states in Nigeria and develop four projection scenarios and estimate death counts to compare how future burden will play out. They conclude that, based on past trends, it is unlikely for Nigeria as a whole, or even for most states therein, to achieve the SDG 3.2 by 2030. And, even if all states achieve unprecedented declines in mortality rate needed to achieve the SDGs, an estimated 6.5 million under-5 deaths would be projected to occur between 2018 and 2030. This paper would be of interest for researchers involved in child mortality estimation and for those working in Nigeria to reduce child mortality.

 The aim of the paper is clear, but I have several comments about the choice of data, the description of methods, presentation of results, and the discussion. I hope the authors will find my thoughts useful as they continue to improve on this work.

Major comments:

1. It is unclear in your methods description which data sources were used for which estimates. You have produced mortality rates for five-year periods (2003-2008, and 2008-2013) and cite the NDHSs of 2003, 2008, and 2013. I would assume you used the 2013 survey for 2008-2013 and the 2008 survey for 2003-2008, but please clarify, because it could also be possible to use the 2013 survey to inform the 2003-2008 period. And in any case, why even mention the 2003 survey if (as I assume, but correct me if I am wrong) you aren’t using it?
2. I am unclear on how mortality rates were assigned for a specific year. You describe a multiple decrement life-table approach for a pooled 10-year estimate from the 2016/2017 MICS. Is this the estimate you call 2017 in tables 4 and 5? Or was this some sort of projection made using the equation from row 3 of table 1?
3. As for that equation (row 3 of table 1), if you are assuming exponential change, shouldn’t you use the following: MR_t = MR_{t-n}*exp(aarc*n), the equation in the last row of table 1. I am not sure when each equation is implemented in your analysis.
4. It’s unclear to me what years were actually used for AARC calculation. Were the estimates from pooled 2003-2008 and 2008-2013 estimates considered 5 years apart from one another? These omitted details have left me a bit confused on exactly how your projected estimates for scenario 2 come about.
5. Instead of pooling 10 years of data, why did you not use a synthetic cohort approach to directly estimate annual mortality rates from the surveys?
6. If I understand your analysis correctly, you are using cohort deaths in year y, instead of period deaths, is this true? Are deaths attributed to a particular year actually deaths for the birth cohort from that year?
7. The 2013 NDHS was designed to allow for representative estimates at the state level. Instead of using geopolitical zone AARCs, why don’t you use estimates from the NDHS to make state level AARCs? Even if you don’t use it for your final model, it would be a very useful sensitivity test to better understand the impact of your simplifying assumption of zone-level AARCs.
8. I was surprised to see that you used a national crude birth rate for projection of births (Supplemental appendix, equation 2). You justify this in a footnote saying that while it will underestimate fertility in the north, it will overestimate it in the south, thus averaging out to make reliable national level estimates. I don’t think this is a justifiable assumption to be making in a paper where your claimed strength is to be making subnational projections. According to the 2013 NDHS, total fertility rates vary over two-fold between states (from around 4 to around 8). This variation is unignorable and will certainly impact your estimates of deaths projected at the state level quite significantly. It should be possible to use the surveys to get subnational fertility to use here.
9. I take some issue with calling an unchanging MR “business as usual”. More realistically, it can be argued that ‘business as usual’ is scenario two (MR declines based on historic trends), and that a flat trend would be exceptionally bad at this point. Beyond just a rhetorical issue, I think this colours your discussion too. It’s really an unrealistic scenario, so then to highlight that the gap between this scenario and the meet-SDG (scenario 4) case is an additional 5 million deaths is needlessly provocative. I think a much fairer “business as usual” comparison would be between scenarios 2 and 4. Still a staggering difference of about 3 million deaths between 2018 and 2030.
10. This paper would be greatly improved if you quantified uncertainty in your estimates of mortality rate and subsequently AARC. As they are derived from population samples, your estimates of mortality are not as precise as is led on in the paper, where you only use mean estimates. For example, without an analysis of the uncertainty, it is unclear if the increases in AARC you observe in the South-East are real. Since you have access to microdata, this should be doable.
11. I was disappointed by the lack of contextual discussion about health in Nigeria. The discussion section was somewhat technical without going into details about the health policies that could be shaping the results presented in the paper. For example, for what reasons is Kwara state possibly doing so well? What policies or interventions have been implemented in the states with the fastest declines? There was a lot of room given to the somewhat obvious conclusion that states could learn from each other’s successes, but the lack of broader context and specific examples seems like a missed opportunity.
12. I hate to be one of *those* reviewers who interject about their own paper, but the lack of mention of Golding *et al.* (2017 ^1^) felt like an oversight. In that analysis, we produced estimates of state level neonatal and under-5 mortality in 46 African countries including in Nigeria. We also estimated the annualized rate of change needed to achieve the SDGs at the subnational level. This felt missing in your ‘Comparison with other estimates’ section. We also found that Nigeria was unlikely to meet the SDGs, but I think it would be very interesting to make direct comparisons between MRs/AROCs from that work and yours. It is also untrue as you state in discussion that yours is the first paper to make subnational projections of NMR and U5MR in Nigeria.

And then I have some more minor comments:

1. In your methods supplement you refer to infant mortality rate (IMR) throughout, but your paper focuses on neonatal mortality. This is a strange disconnect between the two and the supplement should be updated to align with main body of the manuscript.
2. In the intro, you say that the 2016/2017 MICS “for the first time allows state-level estimation of NMR and U5MR”. I believe that the 2013 NDHS was designed to be representative at the state level.
3. The last part of the last paragraph in the discussion section about special-causes and common causes was confusing. I wasn’t sure what this meant.
4. I think figure 2 would be more effective as a map where each state is coloured by the year. The lack of meaning of the X-axis as it currently stands is kind of strange.
5. Table 1, it looks like the rows are mis-numbered as the third row starts back at 1.
6. Table 6, should the last column read “2026 to 2030”?

I confirm that I have read this submission and believe that I have an appropriate level of expertise to confirm that it is of an acceptable scientific standard, however I have significant reservations, as outlined above.

## References

[1] : Mapping under-5 and neonatal mortality in Africa, 2000–15: a baseline analysis for the Sustainable Development Goals. *The Lancet*.2017;390(10108) : 10.1016/S0140-6736(17)31758-0 2171-2182 10.1016/S0140-6736(17)31758-0 PMC568745128958464

# Peer review: Reviewer response for version 1

**DOI:** 10.21956/gatesopenres.14030.r27275

**Published:** 2019-06-18

**Authors:** Abraham A. Salinas-Miranda

**Affiliations:** 1 Center of Excellence in Maternal and Child Health, Department of Community and Family Health, College of Public Health, University of South Florida, Tampa, FL, USA; 2 Centro de Investigaciones y Estudios de la Salud, Universidad Nacional Autonoma de Nicaragua, Managua, Nicaragua

I was asked to review the article entitled "How many child deaths can be averted in Nigeria? Assessing state-level prospects of achieving 2030 sustainable development goals for neonatal and under-five mortality". This article presents projections of neonatal and under-five child mortality in Nigeria under the scenarios of current trends and at best in-country trends. This type of study permits decision-makers to allocate resources for timely action that can accelerate progress toward the attainment of the SDGs. Therefore, it's an important topic and of interest to the readership of the Gates Open Research Journal. The objectives, data sources, methods, and results are consistent and sound. Some minor changes are needed to improve the value of this manuscript. The recommendation is to accept, upon revisions. A summary of my assessment is below:

  

- Abstract: The authors need to indicate the study design in the abstract. There is no mentioning of the overall study design.
- Introduction: The authors did a good job justifying their study and stating the purpose of the study. One aspect needs revision. In the last paragraph of the introduction, the authors mention the unique opportunity of new MICS, but they did not mention the availability of the DHS. Together, these permit the estimation of the scenarios presented. As it is currently written, it could be interpreted that the study was based on MICS only. 
- Methods: The authors presented an adequate account of the selection of data sources, variable definition, calculation of rates, and time horizons. However, they overlooked to indicate the type of study design. It was conducted in three phases, but historical trends were used for the projections. This means that is very likely that the design is either a time series (ecological study) or another form of ecological study that permits trends analysis. To this reviewer, it seems to be an ecological design (given that unit of analysis is not at the individual but at the aggregated level of geopolitical zones), which uses econometric methods (forecasting). 
  In addition, more details are needed about the forecasting technique that was used with STATA as there are several that can be used to estimate the predicted values (e.g. autoregressive (AR) models or autoregressive distributed lag (ADL) models?, jointpoint regression based on permutation tests, ordinary least squares, etc.). This is necessary to permit replicability for other researchers. As it is right now, the arithmetic calculations for the rates are clear, but the statistical explanation is insufficient. The information in the extended data was useful, but it did not contain the information on the statistical program (do.file) used in STATA. 
- Results: These are well-described. Kudos to the authors for using AARD and AARC, as those are more robust measures for trends. 
- Conclusion: The authors support their conclusions adequately based on the results for each of the scenarios presented. The recommendations are reasonable.
  However, the limitations can be expanded to address the issues with using DHS data (cross-sectional, self-reported), not only MICS. They mentioned MICS in the limitations, but not the DHS. Also, they should note the assumptions of forecasting (e.g. the longer the forecast, the less reliable). Trend extrapolation methods that use historical data, such as in this study, use mathematical techniques to extrapolate to the future. The assumption is that the forces responsible for changes in the past, will continue to exert influence in the future. Although this is often a valid assumption when forecasting short term horizons, it may not be for medium and long term forecasts (e.g. year 2043). The further out we attempt to forecast, the less certain we become of the forecast. Hence, the authors need to discuss this limitation for the long term predictions they made under the scenarios proposed. The instability of the sociopolitical environment in Nigeria may need to be discussed as a key factor in determining whether trend extrapolation will be an appropriate forecasting model (projections) in the coming years. For that reason, they should recommend updating the forecasting (projection) in a few years in the future. 
  Nevertheless, despite limitations, the study has value for decision-making as it relies on the most recent and most reliable data sources available for the country of Nigeria and its geopolitical zones.  
- Other: Citations for each of the DHS reports are needed. 

I confirm that I have read this submission and believe that I have an appropriate level of expertise to confirm that it is of an acceptable scientific standard.
